# Cellular and Structural Studies of Eukaryotic Cells by Cryo-Electron Tomography

**DOI:** 10.3390/cells8010057

**Published:** 2019-01-16

**Authors:** Miriam Sarah Weber, Matthias Wojtynek, Ohad Medalia

**Affiliations:** 1 Department of Biochemistry, University of Zürich, 8057 Zürich, Switzerland; m.weber@bioc.uzh.ch (M.S.W.); m.wojtynek@bioc.uzh.ch (M.W.); 2 Department of Biology, Institute of Biochemistry, ETH Zürich, 8093 Zürich, Switzerland; 3 Department of Life Sciences and the National Institute for Biotechnology in the Negev, Ben-Gurion University of the Negev, Beer-Sheva 84120, Israel

**Keywords:** cryo-electron tomography, in situ structure determination, cytoskeleton, nuclear envelope, intermediate filaments, focused ion beam milling, correlative light and electron microscopy, gold nanoparticles

## Abstract

The architecture of protein assemblies and their remodeling during physiological processes is fundamental to cells. Therefore, providing high-resolution snapshots of macromolecular complexes in their native environment is of major importance for understanding the molecular biology of the cell. Cellular structural biology by means of cryo-electron tomography (cryo-ET) offers unique insights into cellular processes at an unprecedented resolution. Recent technological advances have enabled the detection of single impinging electrons and improved the contrast of electron microscopic imaging, thereby significantly increasing the sensitivity and resolution. Moreover, various sample preparation approaches have paved the way to observe every part of a eukaryotic cell, and even multicellular specimens, under the electron beam. Imaging of macromolecular machineries at high resolution directly within their native environment is thereby becoming reality. In this review, we discuss several sample preparation and labeling techniques that allow the visualization and identification of macromolecular assemblies in situ, and demonstrate how these methods have been used to study eukaryotic cellular landscapes.

## 1. Introduction

Understanding the basic mechanisms of life at the molecular level is one of the fundamental aims of cell and structural biology. However, traditional structural biology techniques are restricted to the analysis of macromolecules in isolation, and determine their molecular structure one by one. These studies have yielded an impressive amount of mechanistic information that has revolutionized our understanding of basic processes in cells [1,2]. Nevertheless, cellular processes are usually complex and are not performed by single molecules, but rather by a set of proteins and factors that intimately interact in a concerted manner [3]. During the purification process, the cellular context and many relevant factors are lost. Hence, understanding the architecture of proteins in their native, undisturbed and crowded environment of the cell is fundamental for resolving their functions [3,4]. Since its first application to eukaryotic cells [5], cryo-electron tomography (cryo-ET) has become a pivotal approach in cell biology [6,7,8,9,10,11], microbiology [12,13,14,15,16,17], and virology [18,19,20,21,22], as it is the only available technique that allows structure determination of macromolecular complexes at close-to-native conditions directly within a cell [3,23,24]. Due to recent technological developments, it is becoming possible to acquire a high-resolution snapshot of a molecular process within a cell frozen at a specific point in time [11,25,26]. Advances of other techniques like fluorescence microscopy [27,28] and innovative sample preparation methods [29,30], combined with cryo-ET, offer a unique opportunity to explore native cellular landscapes at unprecedented detail.

In this review, we discuss several applications of cryo-ET to study protein complexes and biological processes in eukaryotic cells. We will focus on different sample preparation approaches that allow the visualization of a variety of cells and cellular compartments. Furthermore, we will explore ways to localize and identify specific elements of interest in the crowded cellular environment.

## 2. Principles of Cryo-Electron Tomography

Cryo-ET is a unique technique capable of yielding a three-dimensional (3D) reconstruction of an object of interest (e.g., organelles, prokaryotes, and intact cells) at a resolution of a few nanometers. Objects of interest are preserved in their native hydrated state, usually by vitrifying the sample within a thin layer of ice, a process called plunge freezing [31,32,33]. Thus, the use of chemicals, fixatives, and detergents can be avoided, retaining the integrity of the cell. Plunge freezing is typically used for samples up to 10 µm in thickness [3]. Therefore, it is well suited to preserve cultured eukaryotic cells and prokaryotes. Thicker samples (<200 µm), such as tissue sections or multicellular organisms like *C. elegans* are usually preserved by high-pressure freezing [34].

**Figure 1:**
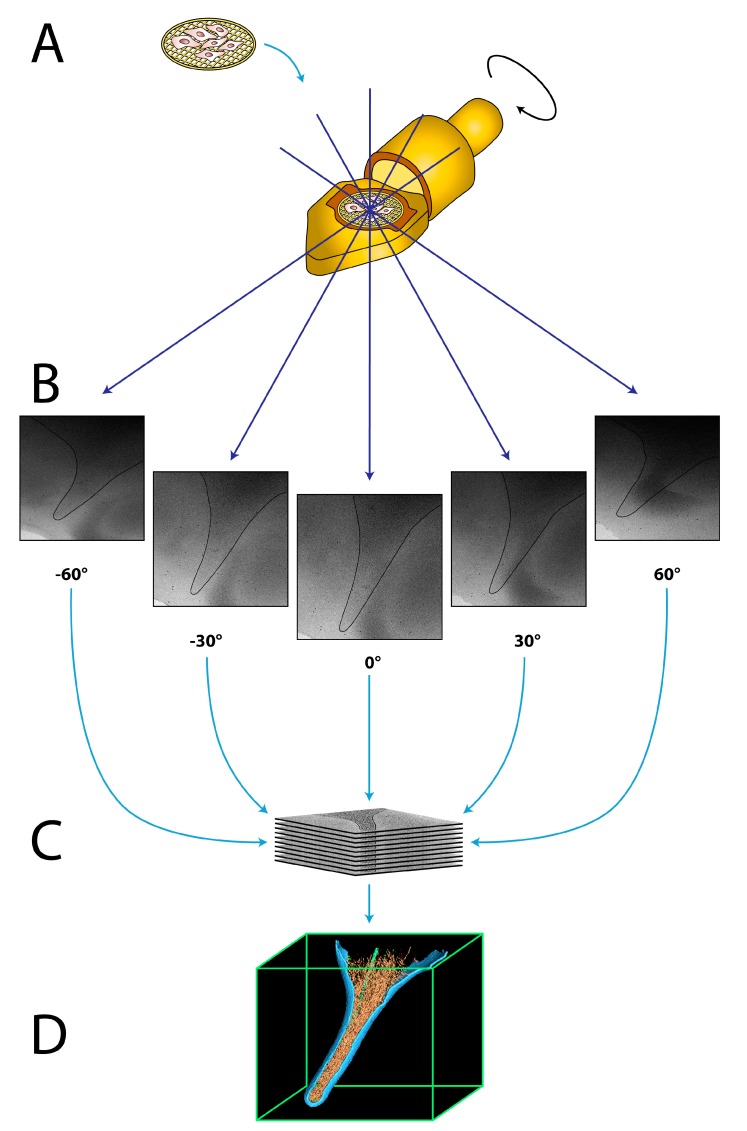
The principle of cryo-electron tomography (cryo-ET). (**A**) The grid containing the vitrified sample is inserted into the cryo-specimen holder of the electron microscope. (**B**) The specimen holder is tilted incrementally around an axis perpendicular to the electron beam, typically from −60 to +60°, while acquiring multiple micrographs. Black line illustrates the plasma membrane of the acquired cell. (**C**) The tilt series is computationally aligned and reconstructed into a 3D density map, a tomogram. (**D**) The 3D tomogram can be inspected and individual components are visualized by surface rendering.

In cryo-ET, multiple two-dimensional projection images of the object are acquired while tilting the sample in the electron microscope, typically between −60° to +60°, in increments of 1° to 4° [35] (Figure 1A,B). The stack of these projection images, termed tilt series, is then computationally aligned to a common feature, typically using fiducial gold nanoparticles, which are added to the sample before vitrification [36]. Accurate alignment is crucial to compensate for movements during tilting of the sample at cryogenic temperatures. Afterwards, the 3D volume of the object is reconstructed into a tomogram, using a variety of well-established algorithms [35,37,38,39] (Figure 1C). The tomogram can be analyzed by visual inspection as well as segmentation of individual components (Figure 1D). In order to retrieve a high-resolution structure of elements of interest, sub-tomogram averaging can be conducted [40,41]. In this procedure, the desired elements are extracted from the tomogram in silico as individual sub-tomograms, which are aligned and averaged together in an iterative process to calculate a highly-resolved 3D structure of the object [41,42]. By averaging multiple copies of the same macromolecules, the poor signal-to-noise ratio of the individual sub-tomograms is greatly improved, and a significantly higher resolution can be obtained. Recent studies have shown that sub-tomogram averaging is capable of resolving structural features to sub-nanometer resolution under favorable conditions [22,43,44,45,46].

One of the major difficulties in unstained cryo-ET of biological samples is low image contrast. As biological specimens consist of mostly light atoms like oxygen, nitrogen, and carbon, contrast formation relies primarily on weak phase contrast [35]. The Volta Phase Plate (VPP), which was introduced by Danev et al. in 2014, is a device that vastly improves the image contrast [47]. The VPP creates phase contrast by introducing a phase difference between the unscattered and scattered electrons that interact with the sample. Thus, the low frequency information, which represents the overall shape of macromolecules, is much better resolved, leading to a substantially improved signal-to-noise ratio. The high contrast of cryo-tomograms acquired with the VPP allows a better interpretation of the observed structures and is therefore highly valuable for imaging of challenging specimens, such as whole cells [10,11,48].

## 3. How to Apply Cryo-ET to Different Parts of Eukaryotic Cells

Cryo-ET is limited by the penetration of electrons through the vitrified sample, restricting the thickness of biological specimens to less than 1 μm [49]. Since most cells are thicker, a variety of sample preparation procedures have been developed to allow imaging of all parts of a cell by cryo-ET. Depending on the localization of the object of interest, different preparation techniques can be employed. Peripheral regions of cells are relatively thin and can be studied in toto, whereas thicker regions need to be thinned before they can be studied under the electron beam. In this section, we will discuss how to image different areas of cells.

### 3.1. Studying Molecular Processes at the Cell Periphery

**Figure 2:**
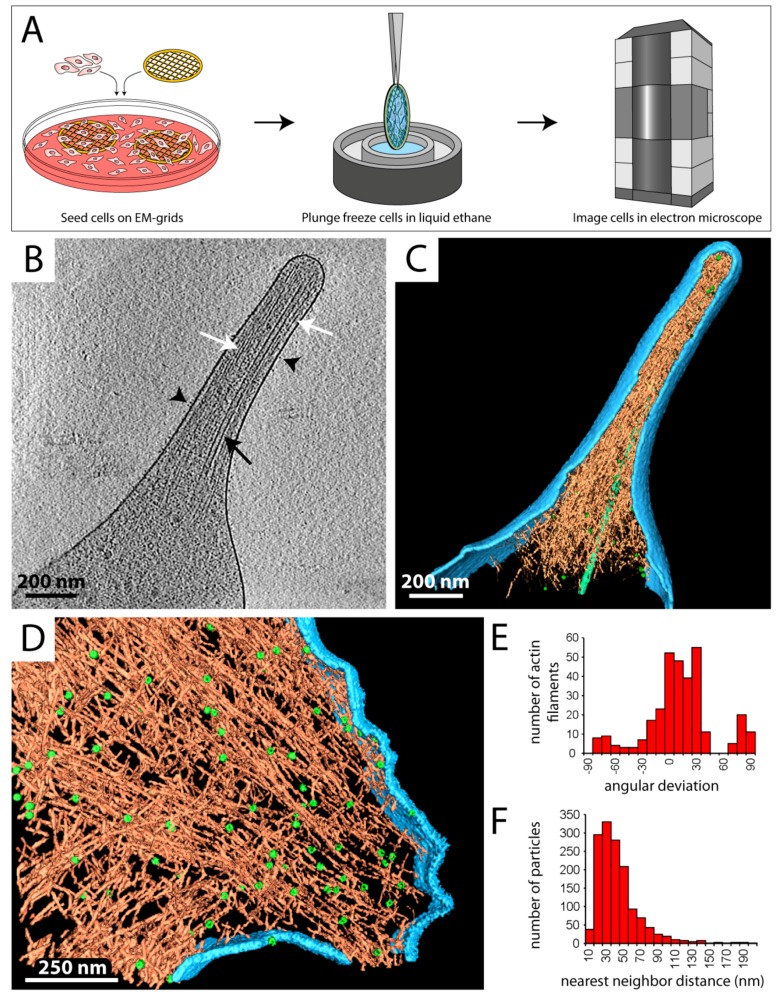
Investigating the cellular periphery by cryo-ET. (**A**) Eukaryotic cells are directly grown or spread on gold EM grids (left), plunge frozen (middle), transferred to the electron microscope, and imaged at cryogenic temperature (right). The cell periphery is subjected to data acquisition by cryo-ET. (**B**) Central x–y slice through a tomogram of a filopodium of a mouse platelet cell. Black arrowheads point at membrane proteins, presumably integrins. Actin filaments (white arrows) and a microtubule (black arrow) are detected in the cytoplasm. (**C**) Surface rendered view of the tomogram in B. Various cellular compartments were computationally segmented and displayed in different colors: plasma membrane (light blue), actin filaments (orange), adhesion-related particles (green spheres), and a microtubule (light green filament). (**D**) Surface rendered tomogram of a focal adhesion site in an ILK^−/−^ mouse embryonic fibroblast (MEF) cell. Coloring according to C. ~90 adhesion complexes and ~320 individual actin filaments could be tracked in this adhesion site. (**E**) Histogram showing the orientation of actin filaments within the focal adhesion site in D. The angular deviation is ~10° larger than in wildtype cells. (**F**) Histogram of the distance between neighboring adhesion complexes versus the total number of particles. (**D**–**F**) are modified from [54].

Spreading and migration of eukaryotic cells rely on the formation of cell protrusions, such as filopodia and lamellipodia. Filopodia are finger-like, actin-rich plasma membrane extensions that protrude at the leading edge of a cell and are involved in early adhesion to the extracellular matrix (ECM), sensing the environment, and cell–cell signaling [50]. Formation of filopodia is driven by polymerization of actin filaments, which are cross-linked into bundles by actin-binding proteins [50,51]. Given their relative thinness (150–400 nm), filopodia are excellent cellular structures for cryo-ET studies, as illustrated in Figure 2B,C.

In this example, mouse platelets were applied to silicon-coated gold EM grids and spread for several minutes, followed by vitrification and analysis by cryo-ET (Figure 2A). Already in the individual 2D projection images, but more pronounced in slices of the tomograms, individual actin filaments (Figure 2B, white arrows) as well as microtubules (Figure 2B, black arrow) can be clearly identified, while on the cell surface transmembrane receptors, presumably α_IIb_β_3_ integrins, are detected (Figure 2B, black arrowheads). These insights enabled a detailed in situ characterization of the native organization of actin filaments within intact filopodia [52,53].

A similar approach was used to study focal adhesion sites and the role of integrin-linked kinase (ILK) in mouse embryonic fibroblast (MEF) cells (Figure 2D–F) [54,55]. Therefore, cells were grown on EM grids overnight, vitrified, and subjected to cryo-ET. As the surface rendering in Figure 2D shows, the length and relative position of individual actin filaments could be tracked, which allowed a structural and quantitative analysis of the actin network in focal adhesion sites (Figure 2E,F). ILK^−/−^ cells showed a major loss of filament alignment (Figure 2E) and an increase in packing density in the adhesion-related particles (Figure 2F) as compared to the wildtype, demonstrating that ILK plays a key role in regulating the focal-adhesion associated cytoskeleton [54].

These examples reflect the strength of cryo-ET in studying molecular processes within an unperturbed, intact cellular environment, an approach that has also been successfully used to study other cellular mechanisms [9,56,57,58]. Additionally, prokaryotes are ideal specimens for cryo-ET studies, as they can often be analyzed in toto. Several studies have yielded remarkable insights into bacterial structures and mechanisms, such as bacterial secretion systems [59,60], motility mechanisms [13,14,61,62,63], and the bacterial cytoskeleton [64].

### 3.2. Deep into the Cell by Cryo-ET

Many processes and cellular structures are localized in the inner regions of a cell, e.g., the cytoplasm or the nucleus, several micrometers away from the plasma membrane. In these regions, the cell is typically very thick (> 1 µm). One of the major drawbacks of cryo-ET is that thick samples are difficult to image, because image formation relies on single scattering of the beam when the electrons penetrate the sample. If the specimen is too thick, the electrons are scattered multiple times when travelling through the sample, which degrades the image quality by increasing the noise. Multi-scattering events increase when the sample is thicker than the mean free path of the electrons, about 350 nm for 300 kV of acceleration voltage [49]. Cryo-scanning transmission electron tomography (CSTET) is a method that allows the analysis of slightly thicker specimens, however it is also limited to ~1 µm thick samples [65]. To make thick specimens accessible for cryo-ET studies, the sample needs to be thinned, typically by physical sectioning of the cell or gentle purification procedures. Here, we will focus on two thinning techniques: focused ion beam (FIB) milling and permeabilization.

#### 3.2.1. Thinning Cells by Cryo-FIB Milling

**Figure 3:**
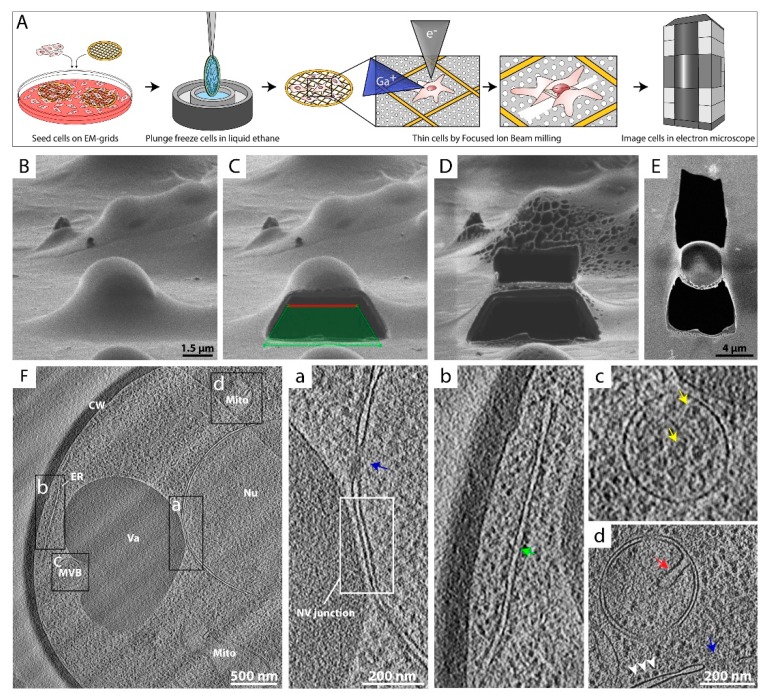
Cryo-focused ion beam (cryo-FIB) milling to analyze structures in situ. (**A**) Sample preparation workflow utilizing FIB milling followed by cryo-ET. Eukaryotic cells are directly grown on or applied to EM grids, vitrified and transferred to a cryo-FIB-scanning electron microscope (cryo-FIB-SEM). A thin lamella of the desired region in the cell is generated by ablating material above and below the position of interest, using a focused beam of gallium ions (Ga^+^) (Scheme was modified from [79]). The progress is monitored by SEM imaging (e^−^). Next, the grid is transferred to a transmission electron microscope for cryo-ET data acquisition. (**B**–**D**) Images of a yeast cell acquired during the FIB milling process from the perspective of the ion beam. (**B**) Intact vitrified yeast cell. (**C**) Yeast cell where the lower part of the cell was removed by the gallium beam. Green tetragon reflects the shape used to direct the gallium beam ablation. (**D**) Remaining ~450 nm thick lamella of the yeast cell. (**E**) SEM image of the produced yeast lamella. Most of the cell volume and the supporting material underneath were removed. (**F**) A 20 nm thick slice through a cryo-tomogram of a FIB-milled yeast cell, acquired at a defocus of −5 µm. Different cellular compartments are labeled: cell wall (CW), mitochondria (Mito), vacuole (Va), endoplasmic reticulum (ER), multi-vesicular body (MVB), and nucleus (Nu). Areas framed black are magnified on the right. (**a**) Close proximity of the nucleus and the vacuole. The interface between the nucleus and the vacuole (framed NV junction) and a nuclear pore complex (NPC, blue arrow) are shown. (**b**) Peripheral ER (green arrow) at the plasma membrane. (**c**) MVB with internal vesicular densities (yellow arrows). (**d**) Mitochondrion with typical cristae (red arrow). In the lower part, ribosomes (white arrowheads) coating the outer nuclear membrane and a NPC pore (blue arrow) are visualized.

To study cytoplasmic complexes or organelles within a eukaryotic cell, a method is required that thins the sample after its preservation in vitreous ice. In previous years, thinning techniques such as sectioning of freeze-hydrated samples with diamond knives have been successfully used to study internal parts of cells or whole tissues by cryo-ET [66,67,68,69,70,71]. However, these techniques have proven to be very challenging and artefact prone [72,73,74,75]. Nowadays, cryo-FIB milling has become a common practice for sample preparation in order to obtain insights into the inner regions of cells [76]. This hybrid method allows the selective production of a thin lamella of a region of interest within thick, fully vitrified biological samples, e.g., cultured cells (Figure 3A). Therefore, cells grown on EM grids are vitrified in their intact, unperturbed state and are thinned using a focused beam of Ga^+^ ions (Figure 3A–D) [29], ablating most of the biological material, until typically a ~100—400 nm thick lamella remains (Figure 3D,E). Cryo-FIB milling followed by cryo-ET and feature tracking-based alignment procedures [36,77,78] have converged to a robust approach to reconstruct the internal organization of bacteria [15,79], eukaryotic cells [8,25], and even multi-cellular organisms [80,81]. Furthermore, the application of sub-tomogram averaging to cryo-ET data acquired from cryo-FIB milled samples allows structure determination in situ at unprecedented resolution [26,82]. 

Figure 3F shows a projection image of a yeast cell that was subjected to cryo-FIB milling followed by cryo-ET, which gives striking insights into the inner landscape of the cell. The architecture of organelles and many of the main cellular compartments can be identified unambiguously. The nucleus with its nuclear pore complexes (NPCs) can be spotted very clearly (Figure 3F), as well as a dark vacuole with a multi-vesicular body adjacent to it (Figure 3F,c). Even the nucleus–vacuole junction is visualized (Figure 3F,a) [83]. A mitochondrion with its typical cristae (Figure 3F,d) and the endoplasmic reticulum (ER) (Figure 3F,b) can be identified as well. The cell is enclosed by the plasma membrane and a thick, electron-dense cell wall.

Recent studies have demonstrated how cryo-FIB can be used to visualize distinct biochemical processes in situ, like vesicle formation at the nuclear membrane during herpes virus maturation [84]. In another study, Albert et al. impressively showed that two distinct structural classes of 26S proteasomes (membrane-tethered and basket-tethered) crowd around NPCs, where they are presumably involved in protein quality control and degradation of misfolded proteins [26]. These and other studies reveal the potential of FIB milling combined with cryo-ET: visualizing individual cellular processes as they occur, at a sufficient resolution that allows direct interpretation of the dynamic processes as well as determination of the structural state of the proteins involved [8,25,82,85,86].

#### 3.2.2. Permeabilization and Minimal-Purification Approaches as Thinning Methods to Image the Nucleus

**Figure 4:**
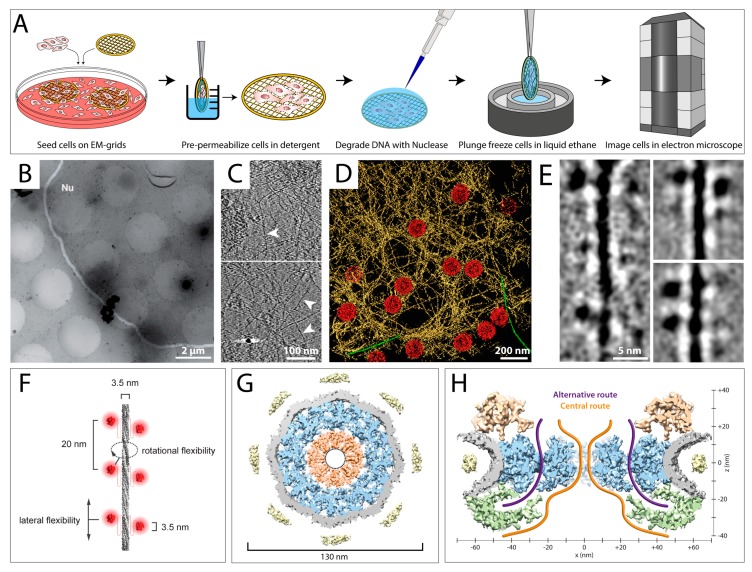
Permeabilization and minimal purification approaches reveal the organization of the mammalian nuclear lamina and the structure of nuclear pore complexes. (**A**) Sample preparation workflow for cryo-ET studies of permeabilized cells. Cells are grown on EM grids, exposed to a detergent for several seconds, and treated with a nuclease to degrade DNA. Next, cells are plunge frozen and analyzed by cryo-ET. (**B**–**F**) Analysis of mammalian lamin filaments in permeabilized MEF cells. (**B**) A low-magnification view of a nucleus within a permeabilized MEF cell. The nuclear membrane is highlighted in white and the nucleoplasm is indicated (Nu). (**C**) Slices through cryo-tomograms of mammalian nuclei. Lamin filaments (white arrowheads) are indicated. (**D**) Surface rendered view of the nuclear envelope, as revealed by cryo-ET of a MEF nucleus. NPCs are colored in red, lamin filaments are colored in yellow. Actin filaments on the cytoplasmic side of the nuclear membrane are shown in green. (**E**) 2D classification of lamin filaments. The Ig-folds can be clearly identified as globular structures, 3.5 nm in diameter, attached to lamin filaments. (**F**) A model of the lamin filaments (grey) and its Ig-folds (red). Distances between the Ig-folds along the filament, the size of individual Ig-folds and the diameter of the lamin filaments are indicated. (**C**–**F**) are modified from [30]. (**G**,**H**) Structural analysis of the *Xenopus laevis* nuclear pore complex. Nuclear envelopes were isolated from *X. laevis* oocytes and spread on EM grids followed by vitrification. (**G**) The central x–y section (10 nm thick) through the NPC shows the organization of the central channel (orange). Substructures are colored as follows: spoke ring (blue), nuclear envelope (grey), and luminal densities (yellow). Inner dotted ring marks masked densities. (**H**) Possible transport routes through the NPC were identified. A 25 nm thick central nucleocytoplasmic section of the NPC with the cytoplasmic side facing upward is shown. The cytoplasmic ring is shown in gold, the nucleoplasmic ring in green. Transport routes are indicated. (**G**,**H**) are modified from [90].

The largest organelle of the cell, the nucleus, is a very dense compartment that cannot be easily studied by FIB milling followed by cryo-ET, as the crowdedness complicates identification of individual proteins. In some cases, the nucleus could be studied by applying a gentle purification treatment [30,87,88,89]. In a permeabilization procedure, cells cultured on EM grids are exposed to a detergent, e.g., Triton X-100, for a short time prior to vitrification (Figure 4A) [30]. The detergent disrupts the plasma membrane and removes most of the soluble components of the cytoplasm, while only insoluble elements such as intermediate filaments (IFs) and the nucleus remain, which can be analyzed by cryo-ET [30]. When residual DNA is of concern, a nuclease treatment can be performed after permeabilization (Figure 4A) [30]. Moreover, labeling approaches can be applied prior to vitrification.

Figure 4B shows an exemplary electron micrograph of a permeabilized MEF cell, where the edges of the nuclear membrane are clearly visible. Figure 4C shows slices through a tomogram of a MEF nucleus at different positions, where individual lamin filaments (white arrowheads) as well as NPCs (white arrows) can be depicted. To allow a better interpretation of the interplay between the lamin meshwork and the NPCs, a rendered view of a nuclear tomogram is shown in Figure 4D. In this study, the lamins could be tracked for long distances and the organization of the mammalian lamina was revealed [30]. Moreover, it allowed studying the structure of individual lamin filaments and uncovered that their diameter is much smaller than had been assumed (3.5 nm instead of 10 nm), supporting the notion that the filaments are formed by tetrameric assemblies, in cross-section [30]. The immunoglobulin-like (Ig)-folds at the C-terminus of lamin dimers could clearly be identified as electron-dense globular structures in 2D classifications of the filaments (Figure 4E). The high resolution of the tomograms even allowed the interpretation of the stacking mechanism of the Ig-folds, giving rise to an improved model of lamin assembly and organization (Figure 4F) [30].

Cryo-ET combined with a gentle purification procedure has also played a major role in structural analysis of the NPC. Due to its large dimensions and its localization in the cell, embedded in the nuclear membrane, the NPC represents an ideal specimen for this treatment [89,90,91]. Figure 4G,H shows a 3D structure of the *Xenopus laevis* NPC, which was reconstructed to 20 Å by sub-tomogram averaging [87]. These insights allowed Eibauer et al. to propose a model of the architecture and gating of the NPC at unprecedented resolution.

## 4. Identifying Structures of Interest within Cellular Cryo-Tomograms

In cryo-ET, the unambiguous identification of a protein or a process in the cell is very challenging, due to the crowded environment and the low signal-to-noise ratio of individual projection images. Furthermore, cryo-tomograms have a limited field of view, typically 3–4 µm^2^, which covers only a small fraction of a classic eukaryotic cell [3]. Therefore, a ‘cellular navigation system’ is of major importance in order to ensure data collection at the desired cellular loci [91]. In the following paragraphs, two methods are described that facilitate the localization of target structures.

### 4.1. Correlative Light and Electron Microscopy as a Tool to Localize Target Proteins

**Figure 5:**
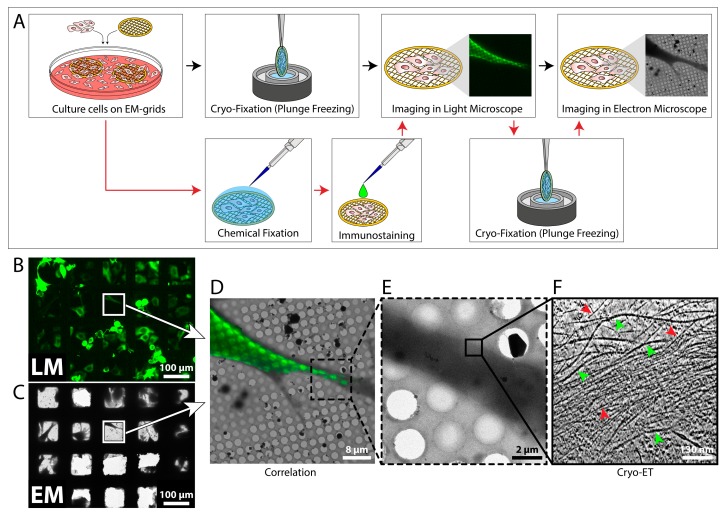
Correlative light and electron microscopy (CLEM). (**A**) Workflow for cryo-CLEM (black arrows) and traditional CLEM (red arrows). For cryo-CLEM, cells cultured on EM grids are vitrified in liquid ethane and imaged in a cryo-light microscope. Next, the grid is transferred to a cryo-electron microscope and the desired positions are found and imaged. Cryo-ET data acquisition is performed at high magnification. For traditional CLEM, cells cultured on grids are chemically fixed and labeled using conventional immunostaining procedures. Next, cells are imaged by fluorescence light microscopy at room temperature, vitrified, and imaged in a cryo-electron microscope. (**B**) MEF cells expressing emerald-vimentin were grown on an EM grid and analyzed by fluorescence microscopy. (**C**) The same region of the grid as in B was identified and imaged in the cryo-electron microscope. (**D**) Overlay of the fluorescence and electron microscopic images of the MEF cell framed in B and C. (**E**) Higher magnification image of the dashed area in D. Cryo-ET data acquisition was conducted at the area marked by the black box. (**F**) A 33 nm thick slice through the cryo-tomogram acquired at the area marked in E. Individual vimentin (green arrowheads) and actin (red arrowheads) filaments are detected in the cytoplasm of the cell.

The localization of fluorescent proteins in cells is a well established tool in cell biology. However, today’s light microscopy (LM) is still limited in terms of spatial resolution and fine structural detail [92,93]. On the other hand, electron microscopy (EM) and cryo-ET can provide sub-nanometer resolution, but with these techniques it is difficult to identify specific processes and objects of interest. Rare events might not be easy to find with the electron microscope, such as subpopulations of cells infected with a virus [91,94]. EM alone is not sufficient to identify these cells, but LM alone might not have the necessary resolution to resolve the studied process [95]. In these cases, correlative light and electron microscopy (CLEM) is a very useful technique, as it combines the strengths of both LM and transmission EM (TEM) and has the potential to elucidate the ultra-structural details of dynamic and rare cellular events [91]. An overview of a CLEM procedure is shown in Figure 5A. Cells are grown on EM grids and can be further processed in two ways: If the protein of interest carries a fluorescent tag, the cells are directly vitrified and imaged in a fluorescence light microscope under cryo-conditions (cryo-CLEM) [95,96,97,98]. Next, the fluorescent image is used as a reference to precisely localize the protein of interest in the electron microscope, and cryo-ET is performed [91,95]. This procedure circumvents the need for chemical fixation and thereby reflects the most native state of the cell [99]. However, cryo-LM is usually restricted to reduced resolution, as commercial cryo-stages for light microscopes are only compatible with air objectives [97]. A variation of the above-described procedure is employed when the protein of interest does not carry a fluorescent tag, or higher magnifications are needed. In this case, the cells are chemically fixed and immuno-labeled, and LM imaging is performed at room temperature (Figure 5A) [28]. Afterwards, the cells are vitrified and analyzed by cryo-ET.

The described procedures may be modified depending on the specimen and structures under examination. For example, when thick regions of a cell are to be analyzed, a permeabilization treatment or FIB milling should be applied before or after LM imaging [96,98]. High-resolution fluorescence imaging prior to FIB milling may even be advantageous in order to guide the milling procedure to the correct location in the cell [96]. Recently, super-resolution cryo-CLEM [100] was realized by analyzing cells with cryo-PALM (photoactivated localization microscopy), which allows a higher degree of correlation accuracy [101,102].

Many studies have already utilized LM in combination with cryo-ET to identify cellular components and study their ultrastructure [94,102,103,104,105,106]. Figure 5B–F shows an example of a CLEM procedure in which vimentin filaments were analyzed. The fluorescent images (Figure 5B,D) were used to guide the tilt series acquisition process to the edge of a cell where vimentin filaments were present (Figure 5B–E). The cryo-tomogram (Figure 5F) contains quantitative information about the vimentin network and the surrounding actin fibers, including length, spacing, and structure.

However, there is still a resolution gap between LM and EM [97]. For super-resolution CLEM, the overall correlation precision currently lies in the range of 50–100 nm [106,107]. In a very crowded environment, it might therefore still be difficult to identify a protein of interest, since a single fluorescent pixel separates into a region with many proteins on the level of cryo-ET [97]. To unambiguously identify a specific protein, labeling it with an electron-dense tag might be necessary.

### 4.2. Gold Nanoparticles as Tags to Identify Target Proteins

**Figure 6:**
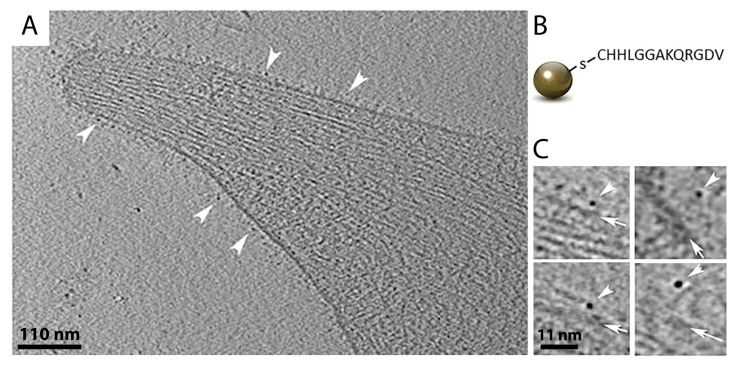
Gold nanoparticles in cryo-ET. (**A**) A 1.2 nm thick slice through a cryo-tomogram of a membrane protrusion of a human platelet cell. Gold nanoparticles bound to integrin receptors on the plasma membrane are indicated by white arrowheads. (**B**) Schematic view of a 2.2 nm gold nanoparticle conjugated to a peptide chain that binds to integrin receptors. (**C**) Gold nanoparticles (white arrowheads) coupled to integrin receptors at the plasma membrane (white arrows) of platelet cells. Figure was modified from [116].

A common way to tag a protein of interest for EM analysis is immuno-gold labeling [108]. Several studies have impressively shown how antibodies coupled to colloidal gold could be used to identify specific proteins by electron microscopy, e.g., to determine the localization of individual components of NPCs or to investigate the co-localization of neurotransmitters in neurons [89,109,110,111,112,113]. In a proof-of-concept study, native immuno-gold labeling was applied to living cells to identify hRSV assembly sites in mammalian cells and study virus–host interactions [114]. More recently, Turgay et al. utilized two antibodies conjugated to colloidal gold nanoparticles in different sizes (6 nm and 10 nm in diameter, respectively) to simultaneously identify lamin A/C and lamin B1 filaments at the nuclear lamina of somatic mammalian cells [30]. This confirmed that the proteins build separate protein meshworks beneath the inner nuclear membrane, as previously shown by super-resolution fluorescence microscopy [115]. However, the disadvantage of using antibodies for protein labeling is that antibodies themselves are quite large (7 nm in diameter), and are additionally coupled to a gold-labeled binding protein, e.g., Protein A or a secondary antibody. This large complex, in combination with the size of colloidal gold itself (typically 5–10 nm in diameter), may create inaccuracy in determining the position of the protein of interest. One potential solution for this problem is the use of smaller gold nanoparticles (~2.2 nm in diameter) that can be directly conjugated to various molecules, such as peptides, proteins, nucleotides, and sugars, which widens their application range enormously [116]. They can be designed as ligands of a specific receptor, or they can be coupled to designed ankyrin repeat proteins (DARPins), which are small antibody homologs that can be generated to bind with high affinity to nearly every protein target [117]. Monolayer-protected gold nanoparticles are relatively easy to synthesize and their small size facilitates a localization very close to the protein structure of interest, making its identification in cryo-ET easier [116]. In a recent study, they were used to precisely locate integrin receptors on the surface of human platelet cells (Figure 6) [116]. Therefore, platelet cells were incubated with gold nanoparticles for 15 min, seeded on EM grids, vitrified, and imaged by cryo-ET. However, in crowded environments or thick samples, slightly larger gold clusters could be advantageous (~3–4 nm in diameter). Similar to gold-coupled antibodies, it is possible to use gold nanoparticles of different sizes and with different affinities to label more than one protein type in a cell. As they can be functionalized to many targets, it is imaginable that future studies will generate fluorescent gold nanoparticles that could combine a precise localization of individual proteins in a cell by cryo-ET with the advantages of a CLEM procedure.

## 5. Conclusion and Perspectives

In situ structural biology is an emerging field aiming at the integration of close-to-atomic precision and cellular context. To date, cryo-ET is the only technique that can be used to obtain high-resolution structures of macromolecular assemblies in the native environment of a cell, thereby combining molecular and cellular structural biology [118,119]. The above described sample preparation approaches and labeling techniques combined with cryo-ET offer a unique opportunity to take a deep look into the molecular life of cells and study their processes at unprecedented detail. The ultimate goal of cellular cryo-ET, however, lies in the integration of several of these techniques. The further development of super-resolution cryo-CLEM to accurately localize structures of interest would allow for the reconstruction of a large number of molecular machines. Combined with phase plate imaging to improve the contrast of the tomograms [47], this could provide high-resolution maps of entire cellular landscapes. Moreover, the integration of several complementary methods, such as atomic force microscopy (AFM) [120,121] and soft x-ray tomography [122], could provide a realistic view of the cell at sub-nanometer resolution in combination with its mechanical properties and overall 3D cellular context.

Albert Einstein once phrased it like this: “Look deep into nature, and then you will understand everything better.” Recent advances in electron microscopy, especially cryo-ET, have enabled us to take a deep look into the molecular life of a single cell and visualize the architecture of nature at unprecedented detail; and indeed, now we understand better.
